# Effect of Self-Determination Theory on Knowledge, Treatment Adherence, and Self-Management of Patients with Maintenance Hemodialysis

**DOI:** 10.1155/2022/1416404

**Published:** 2022-07-20

**Authors:** Rui Wu, Sifang Feng, Hongli Quan, Yun Zhang, Rong Fu, Hong Li

**Affiliations:** Department of Respiratory and Critical Care Medicine, The First Affiliated Hospital of Xi'an Jiaotong University, Xi'an, China

## Abstract

Our aim of this study was to investigate the effect of self-determination theory on awareness of relevant knowledge, treatment compliance, and self-management level in maintenance hemodialysis (MHD) patients. A total of 90 patients who received MHD treatment in our hospital from April 2018 to June 2020 were selected and randomly divided into the intervention group and the routine group with 45 cases in each group. Both groups were given basic hemodialysis patient management measures, and the intervention group was given self-determination theory to manage patients on this basis. The awareness rate of hemodialysis patient-related knowledge, patient treatment compliance, patient self-management scores, and quality of life scores were compared between the two groups before intervention and 6 months after intervention. After intervention, the awareness rate of dialysis principle, reasonable diet, internal fistula protection, and complication prevention knowledge in the intervention group was higher *t* (*P* < 0.05); before intervention, there was no significant difference in the scores of all dimensions of compliance between the two groups (*P* > 0.05); after intervention, the dietary compliance, fluid intake compliance, dialysis regimen compliance, and total score of compliance in the intervention group were higher than those in the conventional group (*P* < 0.05). After intervention, the total scores of problem solving, partnership, emotional processing, self-care activities, and self-management in the intervention group were higher than those in the conventional group, (*P* < 0.05). After intervention, the physical domain, psychological domain, social relationship domain, and total scores of quality of life in the intervention group were higher than those in the conventional group (*P* < 0.05). Self-determination theory management is effective in improving the awareness of hemodialysis-related knowledge, treatment compliance, self-management level, and quality of life in MHD patients.

## 1. Introduction

Maintenance hemodialysis (MHD) is the mainstay of treatment for patients with end-stage renal disease, but patients receiving treatment can easily stop or change doses due to family financial pressures, lack of medical knowledge, and excessive worry about their condition. As a result, medication adherence is poor and quality of life is significantly reduced [1]. Therefore, it has become the focus of medical staff to provide good care for MHD patients, improve their quality of life and self-management ability, make them more aware of nursing knowledge, and improve the health status of patients [2]. In the past, common psychological interventions in clinical practice include cognitive behavioral therapy, social support interventions, relaxation therapy, and physical therapy [3]. Although they can achieve some intervention effect, these interventions do not pay enough attention to the basic psychological needs of patients. The patients in intervention are in a passive acceptance state, and their self-motivation and decision-making ability are difficult to form true self-regulation [4]. Self-determination theory falls within the realm of positive psychology theory, which emphasis the organic interaction between the individual and the social environment. It is clearly stated that only when the three basic psychological needs of personal autonomy, sense of competence, and sense of connectedness are met can the patient maintain a positive therapeutic state [5]. Self-determination theory has been widely used in the population, especially in the fields of education, organization and management, and physical exercise [6]. This study mainly explored the effect of self-determination theory on the awareness rate, treatment compliance, and self-management level of patients with MHD.

## 2. Patients and Methods

### 2.1. Patients

A total of 90 patients who received MHD treatment in our hospital from April 2018 to June 2020 were selected and randomly divided into the intervention group and the routine group with 45 cases in each group. The inclusion criteria were as follows: the age of the patients was 40–75 years old; the patients received regular MHD treatment in our hospital, and the treatment frequency was 2 times a week; the patients with dialysis age were more than 6 months; and the patients have good listening, speaking, and comprehension. The exclusion criteria were as follows: patients with dementia and mental illness; patients with malignant tumors; patients who cannot take care of themselves; patients with other types of diseases that affect the quality of life of patients (cerebrovascular disease, myocardial infarction, and severe); and patients who refused to participate in this study.

Before the implementation of this study, the research plan was submitted to the Medical Ethics Committee of The First Affiliated Hospital of Xi'an Jiaotong University for approval, and it was implemented after the medical ethics committee decided to issue a document (document no. Lunyan (approval) [2017] no. 16). All signed the relevant informed consent. The study was conducted by following the Declaration of Helsinki.

### 2.2. MHD Treatment

A F15 polysulfide membrane hollow fiber dialyzer (Weigao Group, A1EE12100) was used, hemodialysis concentrated bicarbonate (Baxter Medical Products Trading (Shanghai) Co., Ltd.) was used as the dialysate, and the blood flow was set at 220–260 ml/min. The flow rate was set to 500 ml/min. We use low molecular weight heparin sodium (Qilu Pharmaceutical Factory) for anticoagulation, the dose is 50–80 IU/kg, the dialysis time should be 4.0 h/time, 5 times/2 weeks, and the volume of the surface area dialyzer is 1.5 m^2^, the filter. The passing coefficient is 8 ml/(mmHg h).

### 2.3. Interventions

The routine group took the following measures: basic hemodialysis patient management measures were mainly performed by the responsible nurses, and nursing was carried out through instructions and manuals. Educational content included dry weight management, dietary guidance, preservation of vascular access, mental healthcare, healthcare, and more.

The intervention group took the following measures: on the basis of the control group, self-determination theory was also given to manage patients. It was mainly divided into two parts: motivation conversion and needed satisfaction. Motivation transformation: internalize external motivation and be able to decide their behaviors. (1) For unmotivated patients, the patient should be informed of the expected recovery effect, and the difficulties that may be encountered after surgery should be analyzed together with the patient, so that the patient can understand the reasons for the difficulties and discuss the results. Plan and ultimately improve the patient's confidence in rehabilitation, so that patients have the motivation to cooperate with rehabilitation training. (2) For patients who had a motivation for rehabilitation but relied too much on external adjustment, it was informed that postoperative rehabilitation was a long process. In addition to cooperating with medical staff, self-management was also very important. The patient and his family describe the role of self-management in rehabilitation training. We observed the strengths and weaknesses and pointed out that this patient had positive strengths and encouraged them to persevere. We formulated a targeted balanced and reasonable diet plan for patients, so that patients knew how to cooperate with their daily diet to meet their nutritional needs and ultimately achieved the purpose of a reasonable diet. Demand satisfaction as follows: (1) Independent needs: we communicated with patients more, encouraged patients to express their needs to their families or medical staff, and respected patients on the basis of not harming the health interests of patients, so that patients can choose their own activities according to their own wishes. (2) Ability requirements: through one-on-one guidance, knowledge lectures, and distribution of booklets, patient education and ability training are strengthened, so that patients had certain self-care ability, and they used their knowledge and skills to improve patients' confidence in self-management. (3) Relationship requirements: respect patients and establish a harmonious doctor-patient relationship, so that patients can feel the enthusiasm of medical staff and enhance their sense of belonging. We invited patients who had achieved good recovery results to speak up and improved patients' confidence in recovery and encouraged patients to share experience and encourage each other, so that patients can integrate into the group and embrace positive values.

### 2.4. Outcome Measures

The awareness rate of relevant knowledge of hemodialysis patients, treatment compliance, self-management score, and quality of life score were compared between the two groups before and after 6 months of intervention. A self-made questionnaire was used to investigate the awareness rate of hemodialysis-related knowledge, which was mainly investigated from five perspectives: dialysis principle, reasonable diet, regular dialysis, internal fistula protection, and complication prevention to observe whether the patients could correctly answer the relevant knowledge: awareness rate = number of patients correctly answered/sample size in this group.

Compliance was evaluated by the treatment compliance scale for maintenance hemodialysis patients with end-stage renal disease [7], which is mainly evaluated by 23 questionnaire items and 4 dimensions, mainly from four aspects: dietary compliance, fluid intake compliance, dialysis regimen compliance, and medication compliance. The Likert 5-grade scoring method was used for each item, and the better the compliance, the better the score.

The self-management score of patients was evaluated by the self-management scale for maintenance hemodialysis patients developed [8]. The questionnaire had a total of 20 items and 4 dimensions, which were mainly evaluated from four aspects: problem solving, partnership, emotional processing, and performing self-care activities. The Likert 4-level scoring method was used. The score indicated that the better the self-management.

The quality of life score of the World Health Organization (WHO) Quality of Life-Short Form [9] is evaluated from four dimensions: physical domain, psychological domain, social relationship domain, and environmental domain. There are a total of 26 questionnaire items, with a total score of 26–130 points. The score indicates that the better the quality of life of patients.

### 2.5. Statistical Analysis

Statistical Product and Service Solutions (SPSS) 23.0 (IBM, Armonk, NY, USA) was applied for statistical analysis. The independent sample *t*-test was used for comparison between groups for measurement data obeying normal distribution, and the independent sample *t*-test was used for comparison within groups, all expressed as (‾*x* ± *s*). Count data were tested by *χ*^2^ and expressed as rate (%). *P* < 0.05 indicates statistical difference.

## 3. Results

### 3.1. Comparison of General Conditions between the Two Groups

There was no significant difference between the two groups in the baseline data of patients in the conventional intervention group (*P* > 0.05) (Table 1).

### 3.2. Comparison of Awareness Rate of Dialysis-Related Knowledge between the Two Groups

Before intervention, there was no significant difference in the awareness rate of dialysis principle, reasonable diet, regular dialysis, internal fistula protection, and complication prevention knowledge between the two groups (*P* > 0.05); after intervention, the awareness rate of dialysis principle, reasonable diet, internal fistula protection, and complication prevention knowledge in the intervention group was higher than that in the conventional group and the difference was statistically significant (*P* < 0.05) (Table 2).

### 3.3. Comparison of Treatment Compliance Scores before and after Intervention between the Two Groups

Before intervention, there was no significant difference in the scores of all dimensions of compliance between the two groups (*P* > 0.05); after intervention, the dietary compliance, fluid intake compliance, dialysis regimen compliance, and total compliance score of the intervention group were higher than those of the conventional group and the difference was statistically significant (*P* < 0.05) (Table 3).

### 3.4. Comparison of Self-Management Scores before and after Intervention between the Two Groups

Before intervention, there was no significant difference in the scores of all dimensions of self-management between the two groups (*P* > 0.05); after intervention, the total scores of problem solving, partnership, emotional processing, performing self-care activities, and self-management in the intervention group were higher than those in the conventional group and the difference was statistically significant (*P* < 0.05) (Table 4).

### 3.5. Comparison of Quality of Life Scores before and after Intervention between the Two Groups

Before intervention, there was no significant difference in all dimensions and total scores of quality of life between the two groups (*P* > 0.05); after intervention, the physical domain, psychological domain, social relationship domain, and total score of quality of life in the intervention group were higher than those in the conventional group and the difference was statistically significant (*P* < 0.05) (Table 5).

## 4. Discussion

MHD treatment is a long-term process, and patients are in the face of their own disease and physical and mental pain caused by treatment, resulting in anxiety, tension and depression, and other adverse psychological diseases. During MHD treatment, patients often take some drugs according to the actual situation [10, 11]. However, most patients and their families lack knowledge related to the disease and cannot correctly recognize the problem of taking medications during dialysis treatment. It is important that adherence is poor, which seriously affects the prognosis. Studies have shown [12] that the overall level of self-management of MHD patients is very low. By improving the self-management of MHD patients, patients can be promoted to change their bad habits. It improves their own disease monitoring ability, treatment compliance, and subjective motivation, reduces complications, and improves quality of life and survival. Good self-management is an important prerequisite for the timely detection/control and prevention of complications, and patients with higher self-management levels can better adapt to the disease, promote health, and return to society. Therefore, how to improve the self-management level and maintain healthy behavior of MHD patients and improve the quality of life of patients has become an important task for medical staff.

Only by mastering treatment-related knowledge can patients better manage their daily behaviors. Therefore, when formulating nursing measures and interpreting relevant disease knowledge, an easy-to-understand method should be used to enable them to better understand relevant disease knowledge and better use it in daily behavior management [13]. The results of this study showed that after intervention, the dialysis principle, reasonable diet, fistula protection, and awareness of complication prevention knowledge of the patients in the intervention group were significantly higher than those in the conventional group and the differences were statistically significant (*P* < 0.05). These results indicate that self-determination theory helps to improve the awareness rate of MHD treatment-related knowledge of patients and further improve treatment compliance, which is important to improve prognosis. This may be because patients had poor initiative in relevant knowledge during routine care and were a process of passively mastering relevant knowledge. The use of self-determination theory can more actively involve patients in the learning process. Patients are also fully informed about the importance of relevant knowledge. Through group discussion, for example, patients can be motivated to learn and actively participate in processes such as care and rehabilitation. The study results are consistent with existing studies [14]. The compliance of MHD patients is mainly manifested in four aspects: diet, fluid intake, drug therapy, and dialysis regimen. The results of this study showed that after intervention, the dietary compliance, fluid intake compliance, dialysis regimen compliance, and total compliance score of the patients in the intervention group were significantly higher than those in the conventional group and the differences were statistically significant (*P* < 0.05). Self-determination theory can effectively stimulate the adaptability and self-regulation ability of patients and maximize the mobilization of advantageous resources of patients through close cooperation with patients and their families, thereby reducing the impact of negative factors, the burden of self-experience of patients, and negative emotions, making them more willing to follow the guidance of medical staff and greatly improving compliance.

Self-managed MHD patients can help patients correct bad habits and can also improve their ability to monitor disease conditions, treatment compliance, and subjective initiative, thereby reducing complications and improving quality of life [15, 16]. In this study, the total scores of problem-solving, partnership, emotion processing, executive self-care activities, and self-management of patients in the intervention group were higher than those in the conventional group and the differences were statistically significant (*P* < 0.05). It is consistent with the results of existing studies [17]. These results indicate that both nursing interventions can improve the self-management behavior of MHD patients, while nursing interventions guided by self-decision theory can improve the self-management behavior of patients. On the one hand, patients are informed of the importance of self-management throughout the intervention by means of videos, verbal emphasis, and group discussion. Patients are provided with reasons to recognize the importance of daily behavior management and to reduce controlling behavior throughout the intervention. Patients can discuss and communicate with patients' existing problems by listening and jointly develop plans to improve patients' management ability. Patients' emotions can be vent during interviews. In routine care, patients rarely actively seek care providers to ask questions during dialysis. However, nursing interventions based on self-determination theory can make patients gain more attention and support patients' autonomy, and patients' emotions can be more released.

The ultimate goal of rehabilitation interventions for MHD patients is to improve their quality of life, by meeting the patient's autonomous needs, patients feel that decisions are made by themselves, and they are decision-makers rather than forced to manage their behavior, so that patients themselves truly understand the importance of managing their behavior and thus actively rehabilitate [18, 19]. The results of this study showed that after intervention, the physical domain, psychological domain, social relationship domain, and total score of quality of life of patients in the intervention group were higher than those in the conventional group and the differences were statistically significant (*P* < 0.05). The routine care of MHD patients mainly focuses on the dialysis treatment itself, and the nursing effect is also the most obvious improvement in the physical aspects of patients, while ignoring the psychological and social needs of patients, so the effect of improving the quality of care and life is not ideal, and the patients cannot better master the knowledge of rational drug use. Self-determination theory can not only enhance the rehabilitation motivation of patients and stimulate personal decision-making ability but also enable patients to form self-regulation during disease recovery. From passive care to active self-management, it is conducive to the establishment of health behaviors in the process of rehabilitation, thereby improving rehabilitation efficiency and promoting functional recovery; self-determination theory can meet the needs of patients' autonomy, ability, and interpersonal relationship, facilitate individuals to develop in a positive and healthy direction, and ultimately promote the recovery of patients' function and the improvement of quality of life.

Currently, self-determination theory is widely used for self-management behaviors of patients with chronic diseases and is rarely used in the field of care in China. This study can provide a theoretical and practical basis for constructing nursing intervention programs for MHD patients in the future. In this study, a combination of group education and personal guided interview was used to meet the three basic psychological needs of patients for autonomy, ability, and sense of belonging, internalize patient motivation, and improve patients' self-control ability by reducing the control behavior of nursing staff. However, this study is a single-center study protocol, sample selection may have certain deviations, with certain limitations, and then multicenter large-sample study can be used to further explore the application effect of theory in nursing intervention.

## 5. Conclusions

The use of self-determination theory management in MHD patients has a certain effect on improving patients' awareness of hemodialysis-related knowledge, treatment compliance, self-management level, and quality of life.

## Figures and Tables

**Table 1:** Comparison of general conditions between the two groups.

General information	Intervention group (*n* = 45)	Conventional group (*n* = 45)	*t*/*χ*^2^ value	*P* value
Age (years)	63.8 ± 8.2	62.5 ± 8.2	0.752	0.454

BMI (kg/m^2^)	24.1 ± 2.0	23.8 ± 2.0	0.712	0.479

Years of education	10.9 ± 2.5	10.6 ± 2.5	0.569	0.571

Age on dialysis (months)	21.8 ± 5.3	20.9 ± 6.0	0.754	0.453

Sex (%)			0.714	0.398
Male	26 (57.78)	22 (48.89)		
Female	19 (42.22)	23 (51.11)		

Primary disease (%)			1.183	0.757
Glomerulonephritis	26 (57.78)	29 (64.44)		
Diabetic nephropathy	8 (17.78)	9 (20.00)		
Hypertensive nephropathy	7 (15.56)	4 (8.89)		
Others	4 (8.89)	3 (6.67)		

**Table 2:** Comparison of awareness rate of dialysis-related knowledge between the two groups (*n* (%)).

Group	*N*	Dialysis principle		Reasonable diet		Regular dialysis		Fistula protection		Prevention of complications	
		Preintervention	Postintervention	Preintervention	Postintervention	Preintervention	Postintervention	Preintervention	Postintervention	Preintervention	Postintervention
Intervention group	45	26 (57.78)	43 (95.56)	35 (77.78)	45 (100.00)	39 (86.67)	45 (100.00)	32 (71.11)	45 (100.00)	31 (68.89)	43 (95.56)
Conventional group	45	29 (64.44)	33 (73.33)	38 (84.44)	42 (93.33)	41 (91.11)	43 (95.56)	36 (80.00)	38 (84.44)	30 (66.67)	33 (73.33)
Χ^2^ value		0.421	8.459	0.278	3.103	0.45	2.045	0.963	7.590	0.051	8.459
*P* value		0.527	0.004	0.598	0.078	0.502	0.153	0.327	0.006	0.822	0.004

**Table 3:** Comparison of treatment compliance scores before and after intervention between the two groups (‾*x* ± *s*, minutes).

Group	*N*	Dietary compliance		Fluid intake compliance		Dialysis regimen compliance		Medication compliance		Total score	
		Preintervention	Postintervention	Preintervention	Postintervention	Preintervention	Postintervention	Preintervention	Postintervention	Preintervention	Postintervention
Intervention group	45	21.51 ± 4.20	28.64 ± 3.59	12.65 ± 2.77	18.63 ± 2.80	12.65 ± 2.85	18.00 ± 3.17	18.63 ± 3.25	21.70 ± 3.54	68.95 ± 7.80	87.03 ± 8.11
Conventional group	45	20.70 ± 4.63	23.81 ± 4.03	13.80 ± 2.92	15.42 ± 3.11	13.30 ± 3.18	15.74 ± 3.64	17.84 ± 3.41	20.52 ± 3.28	66.30 ± 7.54	75.11 ± 7.83
*t* value		0.869	6.003	1.917	5.146	1.021	3.141	1.125	1.640	1.639	7.093
*P* value		0.387	0.000	0.059	0.000	0.310	0.002	0.264	0.105	0.105	0.000

**Table 4:** Comparison of self-management scores before and after intervention between the two groups (‾*x* ± *s*, minutes).

Group	*N*	Problem resolution		Partnerships		Emotional processing		Self-care activities		Total score	
		Preintervention	Postintervention	Preintervention	Postintervention	Preintervention	Postintervention	Preintervention	Postintervention	Preintervention	Postintervention
Intervention group	45	10.71 ± 2.54	16.23 ± 3.94	9.68 ± 2.70	13.85 ± 3.77	9.54 ± 2.25	15.45 ± 3.11	15.46 ± 3.88	23.64 ± 4.75	46.11 ± 9.50	68.61 ± 9.24
Conventional group	45	11.40 ± 2.72	13.66 ± 3.35	10.31 ± 2.84	11.64 ± 3.54	10.38 ± 2.50	12.75 ± 2.98	16.90 ± 4.21	18.95 ± 4.31	48.67 ± 7.74	55.87 ± 8.80
*t* value		1.244	3.334	1.078	2.867	1.675	4.205	−1.687	4.905	−1.401	6.698
*P* value		0.217	0.001	0.284	0.005	0.097	0.000	0.095	0.000	0.165	0.000

**Table 5:** Comparison of quality of life scores before and after intervention between the two groups (‾*x* ± *s*, minutes).

Group	*N*	Physiological domain		Psychological domain		Social relations domain		Environmental area		Total score	
		Preintervention	Postintervention	Preintervention	Postintervention	Preintervention	Postintervention	Preintervention	Postintervention	Preintervention	Postintervention
Intervention group	45	20.55 ± 4.82	25.64 ± 5.12	21.60 ± 3.95	25.44 ± 4.63	6.93 ± 2.11	9.50 ± 3.08	25.64 ± 4.93	28.23 ± 5.43	73.95 ± 8.53	88.20 ± 8.87
Conventional group	45	21.85 ± 3.90	22.39 ± 4.85	22.33 ± 4.21	22.81 ± 5.02	6.20 ± 1.86	7.54 ± 2.30	26.81 ± 5.28	27.06 ± 5.70	75.67 ± 8.26	79.14 ± 9.35
*t* value		1.407	3.091	0.848	2.583	1.741	3.420	1.086	0.997	0.972	4.716
*P* value		0.163	0.003	0.399	0.011	0.085	0.001	0.280	0.322	0.334	<0.001

## Data Availability

The datasets used and analyzed during the current study are available from the corresponding author upon request.
